# Ethical Implications of e-Health Applications in Early Preventive Healthcare

**DOI:** 10.3389/fgene.2022.902631

**Published:** 2022-07-08

**Authors:** Mandy Stake, Bert Heinrichs

**Affiliations:** ^1^ Institute for Neuroscience and Medicine: Brain and Behaviour (INM-7), Jülich Research Center, Jülich, Germany; ^2^ Institute of Science and Ethics (IWE), University of Bonn, Bonn, Germany

**Keywords:** e-health, AI, pediatrics, preventive health care, early health examinations, ethics, best interest of the child, group benefit

## Abstract

As a means of preventive medicine early detection and prevention examinations can identify and treat possible health disorders or abnormalities from an early age onwards. However, pediatric examinations are often widely spaced, and thus only snapshots of the children’s and adolescents’ developments are obtained. With e-health applications parents and adolescents could record developmental parameters much more frequently and regularly and transmit data directly for ongoing evaluation. AI technologies could be used to search for new and previously unknown patterns. Although e-health applications could improve preventive healthcare, there are serious concerns about the unlimited use of big data in medicine. Such concerns range from general skepticism about big data in medicine to specific challenges and risks in certain medical areas. In this paper, we will focus on preventive health care in pediatrics and explore ethical implications of e-health applications. Specifically, we will address opportunities and risks of app-based data collection and AI-based data evaluation for complementing established early detection and prevention examinations. To this end, we will explore the principle of the best interest of the child. Furthermore, we shall argue that difficult trade-offs need to be made between group benefit on the one hand and individual autonomy and privacy on the other.

## 1 e-Health in Preventive Health Care in General and in Pediatrics in Particular

According to advocates, big data and AI can dramatically improve preventive healthcare, help establish networks linking patients’ experiences and experts’ knowledge, and bridge the gap between research and individual therapy (Ehrich et al., 2018). Yet at the same time, there are serious concerns about the unlimited use of big data in medicine. Such concerns range from general skepticism about big data in medicine to specific challenges and risks in certain medical areas (Summa et al., 2020). In this paper, we will focus on preventive health care in pediatrics and explore ethical implications of e-health applications.^1^ Specifically, we will address opportunities and risks of app-based data collection and AI-based data evaluation for complementing established early detection and prevention examinations. To this end, we will explore the principle of the best interest of the child. Furthermore, we shall argue that difficult trade-offs need to be made between group benefit on the one hand and individual autonomy and privacy on the other.

Big data and AI have long since reached medicine (Yang et al., 2021). This is no more than a truism. Nevertheless, implementation in everyday medical practice is only just beginning and many questions–including ethical ones–are still unanswered. Policymakers are strongly promoting e-health because they see it as a unique opportunity to improve medical care and because they hope to reduce costs in the medium and for long term. A case in point is the European Commission’s *e-Health Action Plan 2012–2020* which describes e-health as a more personalized, targeted healthcare that can be more effective and efficient, while also facilitating equality and patient empowerment (European Commission 2012). In a similar vein, the World Health Organization underlines the important role of digital technologies for the achievement of universal health coverage and for reaching the Sustainable Development Goals (WHO 2011). The hope for better health care does not seem to come out of thin air. Evidence shows that health apps can improve the efficiency and quality of health care while reducing costs (Bates et al., 2018). Ethical concerns must not be ignored, however, but should be taken into account from an early stage on in order to find appropriate solutions that ultimately increase the quality of medical care and perhaps even reduce costs. The high relevance of e-health applications as part of an increased interconnectivity and availability of medical data is supported by a political and social agenda. However, it also points to the interest of other actors, such as app providers and medical institutions, in the health-related data market, seeking potential monetary gains and possibly power through surveillance (Zuboff, 2019; Sadowski, 2020). When data is used as capital, in particular in the medical context, specific ethical concerns arise. Ensuring informational self-determination and data protection is certainly among the greatest challenges of e-health approaches. However, other ethical principles with which medical ethics has long operated should also be considered (Beauchamp and Childress, 2019). An attempt to ethically evaluate e-health applications in the context of big data also needs to bear in mind the political and social dimensions as well as the theoretical concepts of health, disease and normality. Moreover, power relations and interests of particular organizations, corporations, social groups (children, parents, physicians/researchers), and of other stakeholders like politicians or lobbyists are relevant. Again, the commercialization of medical data and the “technocratic power” (Sadowski 2020) over values, social goods and decisions about what ways of data extraction, data gathering, and data evaluation are acceptable, is of critical relevance in this context. In this study, although important, these dimensions can only be addressed on the sideline; they are, however, discussed more thoroughly, for example, in Deborah Lupton’s *Digital Health* (Lupton, 2018) or more recently in Jathan Sadowski’s *Too Smart* (2020).

The idea of using AI in medicine is older than one might think. Discussions about the implementation of AI can be traced back at least to Paycha (1968). In the specific context of pediatrics, one of the first approaches date back to 1984 when Kohachiro Sugiyama and Yasuhiro Hasegawa introduced the computer assisted medical decision-making system SHELP. Despite this history, pediatrics has received comparatively little attention in e-health initiatives so far. One reason for this could be purely practical, as e-health applications are not yet very pediatrician-friendly and require specific knowledge and information technologies that have yet to be deployed (cf. Kokol et al., 2017: 4). This is in line with the typical pattern that an increase of medical knowledge usually first leads to practical improvements for adults and is only later implemented in the field of pediatrics. Another reason could be that medical care of minors always involves special ethical and juridical challenges. Minors are considered a “vulnerable group” for whom particularly high levels of protection apply. However, the status of a vulnerable group can also be used as an argument that medical care needs to be improved particularly urgently. Children and adolescents should certainly not be deprived of possible improvements in medical care out of excessive caution. At first sight, the approach to improve mobile health (m-health) data collection via apps supported by mobile communication devices like mobile phones, tablets, personal digital assistants (PDAs) or smart watches seems to be promising. The data collected by these means could be analyzed in combination with AI algorithms. In fact, there already is a growing number of apps for monitoring children’s health. Caregivers have the choice to use apps for a variety of topics, including infant care issues, mental health information and support, oral health knowledge, diabetes control, asthma monitoring, management of acute pain, overweight management, or oncologic symptom monitoring (cf. Radovic et al., 2016; Alqarni et al., 2018; Chatzakis et al., 2019; Seidman et al., 2019; Hsia et al., 2020; Martínez et al., 2020; Tragomalou et al., 2020). For monitoring development parameters, parents can choose from a number of apps as well. A search in app stores leads to several apps offered by universities, startups or multinational electronics companies with varying ratings, costs, and features. Although 58% of mobile phone users already downloaded a health-related mobile app as of 2015 (Krebs and Duncan 2015) and an ever-increasing demand is being noted (Carroll et al., 2017; Stewart 2021), several reviews show that a huge number of poor-quality apps, especially information apps and tracking apps that parents use for their children, makes choices difficult (Richardson et al., 2019; Virani et al., 2020): The outcome and the quality of apps depend on the task or goal that they were created for. Generally, m-health applications can be (i) used as data collection platforms and (ii) the collected data can be used for informational purposes in medical practice and healthcare. While there are apps that serve only one of these two purposes, in practice they are often intertwined (a point we address below in Section 4). More specifically, these applications also differ in their purpose for or effect on the user: for instance, some apps can influence the user’s choices about what to do (e.g. symptom tracking apps, tracking medication usage), the user’s moods (e.g. mental health apps), or the user’s general experience of the interface with the app (e.g. chatting with bots, tracking certain parameters, checking health status). However, many m-health applications lack reliance for right symptom tracking and evaluation, which opens up the possibility of incorrect diagnosis, but also potentially endangers users by not mirroring and even trivializing a given health problem, as for instance chatbots or so-called “conversational agents” in mental health apps with their repetitive and scripted responses.^2^ Low quality also shows whenever an identification of sources is not available or vague, or when there is a lack of current information which lowers the credibility of the information provided (Richardson et al., 2019). Moreover, the majority of the applications are not tested by official regulatory bodies or a patient community, which should be taken as a reason not to rely on them too much at present. The fact that user groups provide data in an uncontrolled and unsystematic manner would also be problematic and should be seen as a lack of quality. Mobile health app usage has shown to differ largely regarding age, education, and e-health literacy skills (Bol et al., 2018), which again can heavily influence the evaluation of the collected data. Thus, it is important to keep in mind that quality assessment is a necessary step for the implementation of m-health on a broad level.

However, when we think about the possibility to use m-health applications in a controlled way and in collaboration with given in-person early examinations, it could still be particularly promising to complement the screening of children, which is carried out on a regular basis in many countries, with e-health solutions. As a means of preventive medicine, early detection and prevention examinations could thus identify and treat possible health disorders or abnormalities from an early age on. Nevertheless, pediatric examinations are often widely spaced, and thus only snapshots of the children’s and adolescents’ developments are obtained. This is one reason why the amount of data in pediatrics is very limited overall. With the current resources of e-health applications, parents and adolescents could record developmental parameters such as weight, height, social interactivity, language usage, or behavior patterns much more frequently and regularly, and transmit these data for ongoing evaluation. In addition, AI technologies could be used to identify previously unknown correlations which, in turn, could lead to improved diagnosis and treatment.

## 2 Early Detection and Prevention Examinations in Pediatrics

Regular health screenings are an essential component of pediatrics providing important information about children’s and adolescents’ status of health and development, and thus providing early detection of diseases but also cases of neglect, maltreatment, and abuse. Many countries around the world have child health screening programs that provide primary health care, preventive screenings and immunizations. Looking at the European Union, there are such programs, for example, in the Netherlands centrally provided by institutions of the youth health authority, the “Consultatiebureau” (cf. NL Ministry of Health 2022); in Austria (cf. KBGG (2021): § 3; MuKiPass 2002: § 2), and in Germany. For a better understanding of these programs, we describe the situation in Germany in more detail.

In Germany, institutionalized early detection and prevention examinations in pediatrics has existed since 1971. All children are entitled by law for regular screening examinations known as “U-Untersuchungen” (U-examinations) until the age of 18. These examinations serve the early detection of diseases that pose a significant risk to the physical, mental, or psychosocial development of the child and are regulated in the *Guideline of the Federal Joint Committee on the Early Detection of Diseases in Children*, or short: Children’s Guideline (“Kinder-Richtlinie”) (cf. Kinder-Richtlinie 2022: §1 (1), p. 6). They are usually performed by a pediatrician or family doctor and take place at fixed time intervals. The U-examinations include physical examinations as well as assessments of the child’s cognitive, social and emotional competencies, including a variety of parameters depending on the child’s age, as well as a consultation with the parents. In addition, special screenings are conducted for specific diseases. Moreover, a child’s vaccination status is assessed. (cf. BMG 2021). The examination results and vaccination status are registered in a standardized child examination booklet, which contains a removable card so that parents can prove to third parties, such as kindergartens, that their child regularly attends the U-examinations without disclosing confidential information (cf. BMG 2021). However, the screenings fall under the regulatory purview of the states and are only mandatory in some states (e.g., in Bavaria, Hesse, and Baden-Württemberg since 2008/2009),^3^ while voluntary in other states (e.g., in Berlin, Saxony, or North Rhine-Westphalia).^4^

Early preventive health examinations are an important health reporting tool that was designed to gather more relevant medical data in pediatrics. This was a first step to reduce the lack of data that persists in pediatrics overall. But although preventive services for children and adolescents are provided in various forms throughout Europe, the amount of pediatric health data is still limited and scattered, i.e., there are data gaps due to often widely dispersed studies. This fact significantly limits not only pediatric health care, but also pediatric research. To be sure, there are attempts to fill these data gaps by scientific studies and regular repeat surveys. In Germany, the most comprehensive study of this type is the “Study on the Health of Children and Adolescents in Germany” (KiGGS, 2018) conducted by the Robert Koch Institute (RKI). This study is carried out over a period of many years and aims at gaining nationally valid, representative data on the health situation of children and adolescents. In addition, other national and international studies and surveys provide insights into children’s health and development. The Information System of Federal Health Reporting (IS-GBE) provides a constantly growing data pool in the form of an online database (cf. BZgA 2022).

## 3 The Potential of e-Health Applications to Collect Child Health Parameters

As mentioned above, the use of e-health applications is on the rise. Health apps can improve the efficiency and quality of health care while also reducing costs (cf. Bates et al., 2018: 1975–6). In particular, such applications can help to collect and analyze medical data. Therefore, the use of e-health applications in pediatrics seems very appropriate. Many people of today’s parent generation are tech-savvy, which makes the collection and transmission of data via smart phones or internet-based software potentially easy to implement. In general, such e-health approaches offer an opportunity to move away from treatments based purely on pattern-based decision making and summary statistics to more individualized approaches and to make more accurate decisions based on more comprehensive data sets (cf. Mayer-Schönberger and Ingelsson 2017: 428). In pediatrics, this would mean that therapeutic measures for individual children could be initiated much earlier and easier than today. Moreover, such approaches could ensure that priorities for epidemiological and health policy measures are identified and surveyed more quickly and studies on child health in all fields could be intensified (cf. Ehrich et al., 2018: 488). In addition, new ways of data collection would allow for a better monitoring of changes in individual parameters and more regular time intervals. AI technologies could then be used to search for new and previously unknown patterns (cf. Ehrich et al., 2018: 491). Eventually, a new data collection could evolve, such as a “Wikipediatrics” where patients’ experiences and experts’ knowledge ranging from clinical research to care research and individual therapy could be represented (cf. Ehrich et al., 2018: 495). This would be a new way of storing and using knowledge for pediatricians enabling them to quickly look up simple parameters, illness factors, correlations, or diagnosis suggestions. In conclusion, the use of e-health applications in pediatric screening seems to have great potential.

## 4 Ethical Considerations

Regardless of the possible benefits described above, there are serious ethical challenges to be considered. They range from general concerns about big data in medicine to more specific issues related to minors. The idea of using app-based methods to monitor the development of children and adolescents, to use predictive knowledge, to monitor health, and to provide data on development and social status faces difficult trade-offs. In general, there are severe ethical issues concerning data extraction, data usage and data safety which we will come back to in the following section. Yet in particular, questions arise about the best interests of the individual child and his or her informational self-determination: If it turns out that the use is not or not always in the best interest of the individual child, then e-health applications could possibly be justified by reference to a group benefit. In this case, balancing issues would arise. We shall discuss these concerns in turn after the outline of some general problems.

### 4.1 e-Health and Big Data in the Medical Context

e-health, and more specifically: m-health, is part of a big data policy in the medical context promoting unique opportunities and efficient improvements in medical care while reducing costs (Bates et al., 2018). They are introduced as a means to collecting and evaluating additional health data as well as giving advice for preventive measures. Yet, as already mentioned above, ensuring informational self-determination and data protection is among the greatest challenges of this approach.

The m-health applications already available serve different purposes and goals. The large number of these applications shows the economic relevance: Data can be used to generate profits. Yet, any data acquired from or by the user can eventually generate profits. Moreover, there is a fair chance that the possibilities to *understand* procedures and to participate in decision making are even more impaired in the medical context than in other contexts: With regard to Big Data, the various purposes for data usage are diffuse and often mix without clear boundaries so that previously separate areas can merge and link information to a health context that was previously not considered relevant (cf. Summa, 2020: 98; Braun and Dabrock 2016: 326). These merges could arise, for instance, by linking health data to lifestyle choices or social environment data from social media, forums, blogs, or specialized communities (Rüping 2015: 794; Krüger-Brand 2015: A1026f.; Müller and Samerski 2016: A1749). Furthermore, the interconnectivity of the data on platforms and devices can make all personal data potentially health related (Bächle 2019: 48). Given these interconnected structures, the chance that data could be re-identified (even if properly anonymized before) increases, so that in turn breaches in data security can hardly be excluded. This results in an enhanced risk for informational self-determination because such cross-data connections may lead to possible discriminatory factors and individualization based on personal background information, as for instance capital assets, lifestyle, or living situation, affecting predictions, recommendations, therapy suggestions as well as the access to and quality of health care services. Especially data transfers have a higher potential for data transgressions which again can lead to the danger of “surveillance capitalism” (Sharon 2018; Zuboff 2019; Tsakiliotes 2021), lower credibility and lower quality of the provided applications.

But when data is (also) used to generate economic profits, particular ethical concerns arise: Not only could data extraction, especially in the money-spinning medicine market, be another stabilizer of the much-discussed problem of a “digital capitalism” (Sadowski 2020) since personal and sensitive health data could be used as currency to create profit for the app providers. What is more, the content of the data evaluation based on data gathering in large data pools can be exploitative when provided and used by corporate actors (Sadowski 2019), and can breach data safety and personal consent, if passed on to other parties as, for instance, to insurance companies that already use data to assess risks and profits and thus could gain even more regulating power and authority in the private lives of the concerned persons (Sadowski 2020: ch. 6).

As was shown, most citizens–and this applies already to adults–do not have explicit knowledge of how their data is being used and how related decision processes take place (Summa, 2021: 113; Sadowski 2020: thesis 4); this is even less the case for children. Thus, it seems that the pure collection of more health data is not enough to argue for better early preventive health care. To the contrary, the pure collection of data without evaluation is not of any value for the app users and thus does not fairly compensate them, which makes this practice at least ethically questionable, Sadowski would even say “exploitative” (2020; thesis 4). Rather the data and the analysis of the data need to be critically appraised (Brault and Saxena 2021: 514), interpreted and evaluated to be valuable for the individual data provider. However, thus far it is not certain if these are feasible tasks in the context of app-based AI in general. In addition, it is unclear how this would influence and shape the scope for the concept of the child’s best interest.

### 4.2 e-Health Applications and the Best Interest of the Child

The concept of the best interest of a person is complex and encompasses aspects of both physical and psychological well-being. In the context of medical and research ethics, the concept can serve as a normative standard for justifying decisions affecting individuals (e.g., Taylor 2016). While being able to live a self-determined life may be seen as a core element of a person’s best interests, there can also be a conflict between subjective desires and what is objectively best for a person. Self-determination can be viewed as an ideal that consists of the “freedom to think, choose, and act on one’s own life path” (Akbar 2019: 9). This ideal implies that a person’s well-being is expressed, among other things, in living their life as they see fit and has value in the larger context of social well-being and equality (Krutzinna 2022: 129). However, medical needs may sometimes not comply with a person’s wishes in order to serve his or her best interests. Nevertheless, major interventions in the self-determination of adults are today generally rejected as paternalistic. This is to say that the best interest of adults today is usually interpreted in individualistic terms and thus dissolved into self-determination. With children, the situation is more complex. The concept of “best interest” plays a more important role here, as their capacity for self-determination is only gradually developing, so that what is in the child’s best interest cannot generally be identified with the child’s own wishes and ideas, i.e. what lies in their self-interest. Often, fulfilling children’s wishes is clearly not in their best interest.

In determining what is in the best interest of the child, parents or guardians play a key role. They have a wide scope for decision-making, which is, however, limited by objective factors. Especially with young children, parents alone must decide what is best to do. As they grow older, the views of the minors themselves become increasingly important. It can be particularly difficult to resolve the tension between the right to informational self-determination of children on the one hand, and measures to protect the child’s health on the other. At the same time, a parent’s refusal to take medical action may cause harm to a child and therefore be considered a violation of custodial duties and a lack of responsibility. This tension corresponds to the inherent conflict between the basic ethical principles of beneficence (or non-maleficence) on the one hand and autonomy on the other.

A thoughtful understanding of a child’s best interest is presented in a recent paper by Jenny Krutzinna (2022). She argues that “despite a bona fide belief that we are assessing a child as a unique individual, with individual needs, traits and preferences, we continue to make many generalizations and category-based assumptions in determining the child’s best interests.” (Krutzinna 2022: 121) According to Krutzinna, a way out of this oversimplification and categorization of “the child” as a homogenous group is a concept that she calls the “model of the individual child” (MIC) that highlights the individuality and uniqueness of a child. This model does not dismiss universal and group-specific characteristics about and comparisons between children, but complements these approaches with an even more child-specific point of view that takes into account the specific character, background, likes and dislikes of the child who is thus seen as the individual person he/she is. In contrast to other approaches, this focus can help to prevent serious misjudgments about what is in the best interest of a particular child (cf. Krutzinna 2022: 123, 127, 141).

What follows from such an approach for the use of e-health applications for child screening? On the one hand, one could draw the conclusion that the interests of children would be particularly protected and supported by e-health applications in child screening since the main goal of their use is precisely a more individualistic approach based on the individual parameters. However, whether such an individual benefit exists and, if so, how big it is, is yet an open question. On the other hand, there is a further restrictive conclusion, since the feasibility of a child specific screening supported by e-health applications would have to be examined and evaluated for each individual case, i.e. whether this approach would be in the child’s best interest, whether the benefits outweigh the disadvantages, and what the short-, medium- and long-term effects on the child’s informational self-determination are. Such detailed examination would arguably render the use of e-health applications in child screening impossible, because they can only be operated effectively if they are applied on a large scale. There is also reason to fear that the vertical asymmetry between adults and children is initially reinforced by such applications, as children are unlikely to be able to understand how they work and what their benefits are at first. This is certainly especially true for young children and may change with age.

Thus, in order to balance the right to informational self-determination on the one hand and medical needs on the other, as envisioned by the concept of the best interest of the child, we suggest that it is essential to develop age-dependent models that take special account of the vulnerability of children. Whenever possible, children should be involved in the use of apps, and they should have the opportunity to have a say in what data is collected and with whom it is shared, of course depending on age. As they get older, children should be allowed to determine more and more for themselves the extent to which such applications are used. These ethical requirements should already be considered when designing such applications.

If one assumes that the benefit for the individual child is rather small, does this automatically mean that the use of e-health applications for early diagnosis is ethically unjustifiable? This conclusion would be premature, as there are other areas where moderate violations of the best interest of the individual child are justified by an overriding group benefit. Therefore, this line of reasoning will now be examined.

### 4.3 Individual Benefit Versus Group Benefit

Originally, the concept of group benefit was introduced in the context of clinical trials. The difficulties and the extent of inclusion of children in research have been discussed broadly (Binik 2018; Kantin 2020). It was particularly difficult to justify the enrollment of minors according to established standards, at least if no direct benefit for participating children was foreseeable. However, to completely prohibit participation in studies without direct benefit to minors would have significantly impaired pediatric research. A way to avoid this consequence was that under certain conditions, group benefit can be a justification for accepting risk or some harm to individuals. For instance, group benefits can be used in addition to individual child protection to justify mandatory vaccinations for children attending kindergartens or schools (see Summa, 2020: 87; Xafis et al., 2019: 235, 238, 247; Winkler 2017: 27). This is a classic trade-off between security for the many on the one side and autonomy for the individual on the other side. Considering research involving minors, the concept of group benefit allows for more flexible trade-offs in certain situations than the strict consideration of the authenticity of every child (cf. Radenbach 2006; Löschke and Heinrichs, 2015).

In the case of an app-based approach in pediatrics, more comprehensive data collection and data evaluation could also be justified with reference to an overwhelming group benefit. For example, children often continue to receive medications “off-label” and the dosage is often based on the dosage for adults, as reliable data for children is lacking (cf. Summa, 2020: 92; Steinmann et al., 2016: 19; Heinrichs et al., 2016). Furthermore, it has been argued that with the use of apps and digital infrastructure, risks for children could be better captured and lead to more research data and better access to existing knowledge (see e.g. Rüping 2015). Increased initiatives could even promote “deep medicine,” as Eric Topol (2019) suggested. As a concept, deep medicine suggests that AI has the potential to assist physicians in everything they do and to establish a more empathetic and trustful physician-patient-relation that today often suffers because of time-limits. Also, e-health apps, so the argument, could have this assisting quality, which could, in turn, be particularly fruitful in the pediatric context (cf. e.g., Ehrich et al., 2018; Li et al., 2022) and eventually improve individual patient-specific care and research (Morris et al., 2021). All of these points are to a great benefit for the group of children. However, the flip side must also be considered. Although vulnerable groups such as children should not be excluded from research, excessive data collection may violate privacy rights and informational self-determination as has already been pointed out above. In the context of data collection, this primarily relates to the lack of controllability of the flow of information in data-driven medicine and reflects the output orientation of governance and policy, as Patrik Hummel and Matthias Braun (Hummel and Braun, 2020: 1f.) have recently noted. Thus, the concept of group benefit must be applied very mindfully. To gain more clarity, it is useful to list the different stakeholders involved and the potential benefits they might have. There are at least four main groups that need to be distinguished:

(i) researchers and physicians who could benefit from data collection by filling research gaps, finding new associations, enabling even earlier detection and prevention methods, and thus creating better and more individualized treatments;
(ii) (ii.a) individual children and (ii.b) their parents–the data providers–who might not immediately or directly benefit from better treatment options;
(iii) (iii.a) (future) children and (iii.b) their future parents, who are future data providers and could benefit from better treatment options;
(iv) other stakeholders who might profit from the data financially or through power gain, like e.g. insurance companies, corporations, lobbyists, app-providers, etc.

There are at least two further aspects which are to be considered consecutively: (1) the problem of bias that relates to the already addressed issues about data quality, interpretation and classification up above, and (2) the impact of e-health applications on the trust relationship between physician, patient and parents.

(1) In e-health applications for early detection, medical data points would be collected either automatically or manually by users (parents or adolescents themselves). However, recent studies show that the quality and validity of the data sets based on these data points via cell phones or wearable devices such as smart watches are rather poor since they are often unstructured and full of random or systematic errors due to different types of sensors, conditions, or variations in applicability, which make any interpretation or result based on them likely to be biased (cf. Brault and Saxena 2021: 514f). In fact, bias can enter in various forms and at various stages: (i) in the problem definition according to the developed algorithm, (ii) in the social or technical intervention where certain types of data sets can be incomplete, under- or overrepresented, (iii) when the feature selection is unevenly distributed across different groups, (iv) because of the model’s dependency on the data sets, (v) model selection and its accuracy, (vi) design of the user interface and user directory (Brault and Saxena 2021: 515f.). This calls into question the comprehensibility of results as well as of conclusions based on these datasets (McDougall 2019). Furthermore, if cross-sectional data is also collected, conclusions could be even more problematic than only sectional data since it increases the amount of possible errors and incomprehensible conclusions, which not the least raises questions about replicability and reproducibility of the results (Brault and Saxena 2021: 514). There is another aspect to consider with cross-sectional data evaluation: The efficiency of the algorithms of e-health applications relies on “grouping”, i.e., on putting individuals into groups according to group-specific characteristics which is, again, a risk factor for bias. The number of characteristics is increased when cross-data connections are included which, in turn, can promote higher intransparency than it would be the case if cross-data connections would not be used. For example, if 10-year-old Betty’s social competence, psycho-social development, or language competence is not only tracked by manually entered information in a specific medical app, but also automatically by data from her social media usage time, the postings or pictures she likes or comments on, the music she listens to, and the language she uses in the messages she writes, then this would be a case for cross-data connection. Another example would be if an algorithm puts different children in the same group with higher risks to develop a certain disease, say asthma, and generates treatment or help suggestions, only because they are living in a certain area or have a particular social background, which is based on information that comes from multiple app-trackers but is not necessarily comprehensible since the information of the conclusion cannot be deduced and followed back to the particular apps. A third example–a risk if data is used for early detection or prediction of possible diseases or increased health risks–is that a child could be categorized as part of a certain group before a disease has actually manifested. Not only should this knowledge be sufficiently protected from access by others, but it should also be treated as confidential and with care since the mere knowledge about a certain disposition to develop a disease can be harmful and may lead to self-stigmatization. In fact, knowledge about a potential increased risk for a disease or a probability-based prediction for a future medical condition can already decrease the person’s well-being (Bächle 2019: 51f.).

There is controversy about how respective protection measures are or can be implemented in app-based AI-applications. A further critical point of grouping in general is that these groups might not be stable because individuals can move from one group to another quickly depending on new data points. This importantly differs from other forms of grouping supervised by researchers as for instance is the case in medical studies. Ad-hoc groups put together by cross-data connections can be thus more biased and inclusion can be more unfair to individuals than usual data evaluation methods due to automatic or manual inputs (by the user) that are insensitive to the sample size (cf. Brault and Saxena 2021: 514). Note that it can be difficult to notice unfair or harmful grouping (cf. Mittelstadt 2017: 481).

Then again, not only the linkage or reconnection of data, but also the decoupling of data can lead to problems: algorithms for data evaluation can also decouple the presence of traditional disease symptoms from medical diagnosis and then be a hindrance for appropriate recommendations and measurements. One common consequence of these issues is that under- or overtreatment is likely to occur based on e-health applications since their conclusions are likely jeopardized by bias issues.

All this shows that there are many ways in which cross data connections gathered by e-health applications can lead to “informational harm” (Richter and Buyx 2016: 316). Informational harm refers to the occurrence and dependence of highly questionable results based on biased algorithms, which may result in over-, under- or other forms of mistreatment. Informational harm can also include the risk of information loss and discrimination, which is especially problematic for people who already belong to vulnerable groups, as is the case of children. Therefore, data collection and recommendations for preventive measures based on data sets may create an increased risk for mistreatment and incorrect decisions, especially for members of groups considered most vulnerable (Braun et al., 2021: 3). Only if the data is evaluated by trained physicians in collaboration with medical informatics and data scientists, it seems reasonable to expect an improvement of medical preventive care (cf. Daniel et al., 2019; Durán and Jongsma 2021).

(2) e-health applications can only be successfully implemented if pediatricians as well as parents and children have confidence in and can rely on their safety and efficiency (cf. Bates et al., 2018: 1975–6). What is more, the reliance on safety and efficiency is also likely to have an effect on the doctor-patient relationship, where trust is a central element. In pediatrics, the relationship and decision-making processes are more complex because three parties are involved: the minor patient, the physician, and the parents. Usually, children trust their parents in making the right decision for them, while trust towards the physician has to be built up. An essential factor for this is the parents’ trust in the doctor. Another factor that can contribute to the child’s trust in the doctor in the long term is habituation during visits, in particular during the regular check-ups described above. However, the relationship of trust can be disturbed, especially if children or their parents have the impression that the child’s interest is not paramount. E-health applications could fuel such an impression if data collection and use are not transparent. The providers of e-health applications, as for instance, the commercial companies developing the apps, the data storage and integration centers, but also the app interface itself, can have a mediating role in the traditional relationship between patients and physicians. This can have positive effects, like the support for the physician via accessibility of data, or recommendations based on data, or the immediate support and help for the app user. However, it can also compromise this relationship because trust between the parties is waning. Considering the data collection practice via apps and the use of interpretation of whole data sets based on collected data points, a lack of transparency in one of the factors could undermine trust in the physician: On the side of the patients, trust in the physicians is partly based on their know-how and understanding of the recommended applications, but also on the usage, sharing, accessibility and confidentiality of the output-information which is not provided directly by the familiar physician but rather by an accessible medical platform. As has been discussed above, new forms of data connection and sharing can easily threaten data safety, but also software viruses and hacker attacks can breach this safety. When sensitive health data is leaked because it was not protected by multiple security levels, this increases the risks for re-identification and possible discriminatory practices.

This indicates that the extensive use of individual data to advance medical knowledge for the benefit of patients may result in today’s patients and their parents having less trust in physicians. First and foremost, this applies to the trust between the engaged adults, i.e. the physician and the parents. The potential data safety might not be a fundamental concern in the relationship between the children and the physician since theirs is more based on the perceived goodwill of the physician towards the children. This might, however, change when children get more awareness and start to use the provided e-health application for self-tracking at some point. Here, understanding and informed consent have to be considered more thoroughly since the awareness about potential conflicts and effects of e-health applications has an influence on the level of understanding, which is necessary to consent to that praxis in a meaningful and informed way. If parents and/or children do not understand the praxis, consent is not informed. In this case, however, the decision on whether the use of e-health applications is in the best interest of the child has to be reconsidered. This is even more the case, if parents or older children would *only* rely on e-health applications without a physician to evaluate the output–which is an increased danger if e-health applications are incorporated in every-day use, and not in relation to the clinical context. Thus, extensive use of individual data to advance medical knowledge for the benefits of patients now and in future could result in today’s (child) patients and their parents, having less trust in physicians and thinking that individual well-being is not the primary concern. This could have an overall negative effect and harm both current and future children, which is important to consider when weighing group benefits. In this light, it seems to make only limited sense to justify the app-based collection of medical data by referring to group benefit.

Note that although there already are data protection concepts developed by data integration centers (cf. Prasser et al., 2018: e57–e65; Mansmann et al., 2020: 30), it is often still unclear how the volume and heterogeneity of the data is to be evaluated and used specifically since the necessary theoretical knowledge and standards for meaningful validation, analyzing, and interpretation of the data is still lacking to create useful infrastructures in the medical context (cf. Krüger-Brand 2015: A1026f.). Health-related data generated by and saved in more secure and regulated environments using laboratory information systems (LIS) differs from other (commercial) self-tracking apps or devices; however the ethical challenges seem to be related to similar issues with only more or less severity: informed consent, privacy, control over personal data and the interpreted output based on the given data (Bächle 2019: 49).

Making sense of data is a complex process in which multiple stakeholders are involved (Neff et al., 2017): we need more comprehensive critical data studies, take technical critiques as a way to actively discuss and contribute to the betterment of these app, and thus improve outcomes by tackling the challenges in an interdisciplinary way, bringing together, scientific and practical knowledge, but also taking into account social dimensions and ethical expertise.

In summary, then, group benefit is a relevant ethical concept that is partially suitable for justifying measures that commit individuals to the benefit of a group or society as a whole. Especially in pediatrics, this concept can be used to justify interventions. It is, however, important to ensure that recourse to group benefit in the discussions does not disrupt trust between physicians, patients, and parents. In addition, the true group benefit must be carefully examined and biases, to which data obtained through app-based applications is particularly susceptible, must be minimized. Otherwise, group benefit could easily turn into group harm.

## 5 Outlook

Lindsey Knake (2020: 2) recently raised the question “Are we ready for AI in pediatrics?” and answered it herself with “not completely”. In this paper, we have specifically discussed ethical implications of e-health applications in early preventive healthcare. We agree with Knake that there are still many challenges that have to be overcome. We further agree with the assessment that existing e-health applications are not readily transferable to the pediatric setting (Kelly et al., 2021: 1). According to our analysis, these challenges revolve around familiar questions about tradeoffs between public benefit on the one hand and individual autonomy, privacy, and freedom of choice on the other. In pediatrics, however, these trade-offs are even more problematic because children belong to a particularly vulnerable group that must be treated with special care and attention. In addition, the physician-patient relationship is more complex in the case of children because parents are involved as another party. It is also important to bear in mind that this is a dynamic relationship in which children mature more and more into self-determined individuals whose ideas become more essential and significant for any decisions in their best interest as they grow older.

Since one of the main reasons for decreased trust seem to be transparency and data safety issues, we need to ensure increased insight in and education about e-health, access rights, and social and legal regulations for patients, parents, care holders, physicians and partaking other stakeholders like app-providers, data platforms etc. Moreover, a comprehensive data ethics needs to provide a framework so that data usage is based on the principles of autonomy, beneficence, non-maleficence, and equal and just access (Summa, 2020: 86 and Xafis et al., 2019: 235, 245). Furthermore, the quality of data and algorithms must be assessed thoroughly. Brault and Saxena (2021: 516f.) have recently highlighted the need of a catalogue of bias, the development of methodological standards for the use of big data and AI in the medical context encompassing the principle of explicability and an ethical sense of accountability, but also the development of a critical appraisal of AI and big data in medicine. This call can only be stressed with regard to pediatrics. In addition, Clémence Pinel et al. (2020) have addressed the contextual social embedment and relational features of data (that are “never raw”) and shed light on how to do “data work” more carefully to contribute in the knowledge production.

These are all important ways to make data usage–and in the long run potentially also e-health applications–more meaningful, valuable and ready for use in the pediatric context and to take advantages of the benefits addressed above. This means, the given challenges should not distract from the fact that the collection of data via e-health applications is potentially beneficial in pediatrics. Established preventive health screenings, as for instance the German U-examinations, could indeed be improved through such applications, to the benefit of both individual patients and pediatrics as a whole. One drawback of this point, however, is that in practice not all potential benefits may materialize immediately, but only in the medium and long term, since sufficient data must first be collected and carefully evaluated. Yet, if implemented successfully, such initiatives can be extended not only to the national level, but also to the European or international level. The biggest risk discussed here was that poor data quality and excessive euphoria about technology will lead to exactly the opposite case, namely that e-health applications could hinder or worsen the established health screenings. In the worst case, the use of e-health applications could generate biased data on the basis of which poor decisions are made, and at the same time damage the trust between physicians, child patients, and parents. There have been few analyses of these groups’ priorities, and comprehensive data on possible ethical, legal, and social facilitators and barriers for the implementation of these new technological means for pediatricians, parents, and children so far remains scarce: for instance, there are some general remarks for parent’s perceptions about mobile technology use of preschool aged children (Genc 2014), and some for the perceptions of young children’s, parents’ and industry stakeholders’ criteria for selecting apps (Dias and Brito 2021). Therefore, preferences, wishes and ideas in the context of e-health applications should be investigated and evaluated in future research. The best interest of the child must remain the overriding ethical principle guiding trade-offs in individual cases. Comprehensive information for parents and children must ensure that the right to self-determination is respected at all times. If this is the case, then e-health applications can be jointly developed and implemented and improve health care in pediatrics in the long term.

## Data Availability

The original contributions presented in the study are included in the article, further inquiries can be directed to the corresponding author.

## Footnotes

1 For the sake of simplicity, we will refer to e-health in what follows, even though this category is broad and not very specific. We use e-health as an umbrella term for a wide range of means for data collection and data analysis. In particular, this includes so-called mobile health (m-health) applications as well as deep learning technologies for searching for unknown patterns.

2 According to preliminary evidence, chatbots have been found to be potentially beneficial, enjoyable and helpful when connected to proper research and in-person treatment; yet the study reviews are highly heterogenic and more research with standardized outcomes is required for a proper assessment (Vaidyam et al., 2019).

3 In these states, laws ensure participation in screenings through data transmission from the registration authorities and pediatricians. If the screenings do not take place, a written invitation is sent, and if this is not followed, the youth or health office can be informed.

4 However, some of these states now have more far-reaching structures, as for example North Rhine-Westphalia: The notification procedure may provide the local public youth welfare agency with additional indications as to whether and which families may need support services to ensure the best interests of the child (cf. UTeilnahmeDatVO: § 1 (2)). The data can be provided by the physician who performed a health examination in a secured written form via secured data transmission channels to the *North Rhine-Westphalia State Center for Health*. If necessary, the latter may inform the local public youth welfare organizations (cf. UTeilnahmeDatVO: §§ 2–4). Regardless whether or not screenings are mandatory or voluntary, there is a country-wide free reminder service provided by the *German Association of Pediatricians and Adolescents* (*Berufsverband der Kinder-und Jugendärzte e. V*.; short: BVKJ) in order to help parents keeping their children’s vaccination and screening appointments in good time by e-mail. Independently of the U-examinations, there are compulsory school entry examinations in all German states.
